# Effect of Feed on the Growth Performance, Nutrition Content and Cost of Raising the Field Cricket (*Gryllus madagascarensis*) as a Sustainable Nutrient Source in Madagascar

**DOI:** 10.3390/foods13193139

**Published:** 2024-09-30

**Authors:** Henlay J. O. Magara, Sylvain Hugel, Brian L. Fisher

**Affiliations:** 1 Department of Feed Development, Madagascar Biodiversity Center, Antananarivo 101, Madagascar; hugels@inci-cnrs.unistra.fr (S.H.); bfisher@calacademy.org (B.L.F.); 2 Institut des Neurosciences Cellulaires et Intégratives, UPR 3212 CNRS-Université de Strasbourg, 67087 Strasbourg, France; 3 Department of Entomology, California Academy of Sciences, 55 Music Concourse Drive, San Francisco, CA 94118, USA

**Keywords:** cricket feed, formulated feed, *Gryllus madagascarensis*, minerals, fatty acids

## Abstract

The field cricket, *Gryllus madagascarensis*, is a sustainable and nutritious food resource that has the potential to mitigate global malnutrition. Feeds provided to this cricket can influence its growth parameters, nutritional content, and the cost of raising it for food. The current study aimed to evaluate the effects of feeds formulated from weeds, agro-byproducts, and chicken feed (control) on the growth parameters and nutritional content of *G. madagascarensis*. The formulated feeds included CFB (25.0% protein), CFC (24.5% protein), CFD (24.0% protein), CFE (23.5% protein), CFF (22.5% protein), CFG (21.5% protein), CFH (20.0% protein), CFI (14.5% protein), and CFJ (13.5% protein), and chicken feed (CFA) (28% protein) was used as the control. The formulation of the feeds was based on the acceptability and protein content of the 12 selected weeds and agro-byproducts. Proximate, mineral, and fatty acid analyses were conducted to determine the nutrient content of each feed, as well as the crickets raised on these feeds. The fastest development time was recorded with CFE and CFC. The highest survivorship (98%) was observed in CFG, CFE, and CFC. The highest body mass (1.15 g) and body length (26.80 mm) were observed in crickets fed CFG. By comparison, crickets fed control feed averaged a body mass of 0.81 g and a body length of 23.55 mm. The feed conversion ratio for *G. madagascarensis* fed CFG, CFE, and CFC was 1.71. Crickets raised on CFH and CFG had the lowest cost of feeding per kg live mass gain. Crickets fed on CFF had the highest quantity of protein (67%), followed by those fed CFG (65% protein); crickets with the lowest protein content (50%) were fed CFJ. Crickets fed on CFG had the highest mineral content. Linoleic acid, oleic acid, and palmitic acid were the major fatty acids. The findings indicate that formulated feeds from weeds and agro-byproducts have great potential to be used as an alternative feed source for crickets for two reasons: their capacity to positively influence the biology and nutrition of the cricket, and they can serve as an inexpensive replacement for chicken feed.

## 1. Introduction

Sustainable food systems improving nutrition and reducing malnutrition are urgently needed to meet the surging demand for protein sources around the world. One such sustainable food system is the farming and consumption of crickets [1,2]. Crickets are considered an alternative and sustainable food because they have a short life cycle, are prolific, and require far smaller quantities of water, feed, and space to raise than conventional livestock [3,4]. Moreover, farming crickets have been reported to cause less environmental damage than livestock, as they release fewer greenhouse gases [5]. Additionally, crickets are reported to be more nutritious than livestock [6].

Edible crickets have been a major source of nutrients for millions of people globally; over 60 species have been identified as being excellent human foods [7,8,9,10]. In Madagascar, the *G. madagascarensis* cricket is consumed as a nutrient-rich food resource [11]. *Gryllus madagascarensis* is a brown field cricket found in and around grasslands and farmlands in the high plateau of Madagascar. Both sexes of this cricket have a short lifecycle and are highly nutritious, making the species a suitable candidate for farming. Although this cricket could represent a potential source of nutrients that can help address the food insecurity, malnutrition, and poverty in Madagascar’s households, its farming faces a plethora of challenges including a lack of identified cricket feeds. Farmers also lack information on how different feeds used in rearing crickets impact the biology of *G. madagascarensis*. Moreover, cricket producers and consumers lack details on the nutrition content of *G. madagascarensis* fed on different feeds and how they compare with the required allowable intake to mitigate malnutrition in Madagascar. These questions form the basis of our study.

The type of feed used has been reported to be a key environmental factor influencing the population growth and nutrition content of farmed crickets [12,13,14,15]. To make cricket farming affordable, farmers require cheap, accessible, and sustainable feeds [16]. In Madagascar, chicken feed is the main feed source used to farm crickets [11,17]. Farmers use chicken feed because of its high protein content, balanced essential amino acids (EAA), abundant essential fatty acids (EFA), range of minerals, and easily digestible energy [18] The chicken feed used by the farmers to feed crickets is usually expensive and unsustainable [19,20]. Chicken feed is expensive due to the high cost of the protein-rich soybeans and fish meal, which are commonly added to other ingredients to make chicken feed [21]. The rising cost of chicken feed, combined with uncertainty in the availability of supplies, has forced cricket farmers and feed manufacturers to seek its replacement in inexpensive, abundant, readily available alternative feed sources.

Weeds and agro-byproducts have been proposed as promising alternative materials for formulating cricket feeds [15]. The main advantages of using weeds and agro-byproducts for formulating cricket feed are their local availability, abundance, inexpensive nature, sustainability, and capacity to provide nutrients for crickets [18,20]. Several studies have reported on the potential use of crop residues and weeds as single cricket feeds [22]. However, no previous research has attempted to formulate feeds for edible crickets in Madagascar from weeds and agro-byproducts, making this the first study. From this perspective, formulating a reliable, robust, and sustainable feed requires a clear scientific understanding of the effect of different formulated feeds on cricket growth, survival, reproduction, and feed utilization. Moreover, the nutrient composition of the developed feeds and their impact on the nutrient content of crickets need to be unraveled. Thus, our study aimed to determine the development, survival, growth, reproduction, feed consumption rates, and feed conversation ratios of *G. madagascarensis* fed on various feeds formulated from weeds and agro-byproducts available in central Madagascar. In addition, this study compared the nutrition content of the various formulated feeds with chicken feed and the nutrition content of the crickets fed on each feed. Lastly, this study compared the cost of each formulated feed to chicken feed. The best-performing feed regarding cricket nutrition, availability, and cost is recommended for cricket farming across Madagascar, and more generally, the African mainland, where the weeds and agro-byproducts studied thrive.

## 2. Materials and Methods

### 2.1. Study Insects

The *G. madagascarensis* crickets used for this study were obtained from laboratory populations at the Madagascar Biodiversity Center, Antananarivo (18.9326° S, 47.5254° E; approximately 1280 m above sea level). *Gryllus madagascarensis* is a brown field cricket farmed in Madagascar. It was selected for this study because it is a native cricket with a short lifecycle, is prolific and hardy, and is consumed by Malagasy people. The experiments were conducted using nymphs that were one day old before being provided any feed.

### 2.2. Source of Experimental Feed and Their Processing

A total of 11 plant-based product powders, made from tropical African morning glory, cassava leaf tops, silver leaf *Desmodium*, maize bran, wheat bran, cow pea bran, navy bean bran, gallant soldier, taro leaves, rice bran, and cassava tuber bran were used to formulate experimental feeds. The plant-based products used for feed formulation were chosen based on their acceptability and impact on the biology of the cricket in our previous work [23]. The reference feed used for this study was chicken feed used in cricket research and cricket production farms in Madagascar. Cowpea bran, navy bean bran, maize bran, wheat bran, rice bran, and cassava tuber bran were purchased from a vendor at a local market (Mamahasina market, Antananarivo). Tropical African morning glory, cassava leaf tops, taro leaves, silver leaf *Desmodium,* and gallant soldier were obtained from organic farms and the wild in central Madagascar. They were cleaned using tap water and dried in the sun for 24 h at an average temperature of 30 ± 2 °C. The selected plant-based products then were milled into powders (0.02 mm diameter) using an electric grinder installed at the Madagascar Biodiversity Center (locally fabricated in Madagascar). Due to variations in the nutritional content of the weeds and agro-byproducts, the single-plant products in each diet were not constant (Table 1).

### 2.3. Feed Formulations

The 11 single-plant products most accepted by *G. madagascarensis* from our previous study were used to develop nine different feeds [23]. Each feed was formulated into one kilogram. The formulated feeds were made by combining two to seven ingredients. For example, two ingredients were used to make one feed, three ingredients were used to make a second feed, four ingredients were used to make a third feed, etc., until seven ingredients were used to make feeds based on their level of acceptance by *G. madagascarensis* (Table 1). The combinations were made by considering the nutrition content of the selected single-plant products, resulting in blended feeds with different protein, carbohydrate, fat, and mineral contents. The nutrition content of these feeds in turn influenced the biology and nutrition content of the cricket, allowing the determination of the best feed (Table 2). The nine feeds developed were CFB, CFC, CFD, CFF, CFG, CFH, CFI, and CFJ (Table 1). The feeds developed were then kept in Ziploc bags and stored on a shelf with a closable door at room temperature. The chicken feed (CFA) developed by and purchased from LFL Feed Company, Antananarivo, Madagascar, was used as a control.

### 2.4. Experimental Design

A completely randomized design was carried out using CFA (the chicken feed used as a reference feed) and nine formulated feeds: CFB, CFC, CFD, CFE, CFF, CFG, CFH, CFI, and CFJ (Table 1). The experiments were set in a laboratory with the following environmental conditions: 30 ± 1 °C temperature; 50–60% relative humidity; and 12 h of light: 12 h of dark day period. Temperature and relative humidity were recorded using a thermal hygrometer data logger. During the experiment, single, newly hatched *G. madagascarensis* juveniles were raised in plastic containers measuring 17 cm long by 11 cm wide and 7 cm high with ventilation holes in the lids covered with wire mesh. Experiments on each feed were replicated 150 times (whereby each single cricket in a container was treated as a replicate). The cages were placed on shelves raised 0.5 m above the floor to prevent the bias effect from low temperatures on the floor. Cages containing experimental insects were put 10 cm from the wall of the room housing the experiment to prevent bias from the wall effect. Cages were also placed 5 cm away from each other to avoid cage effect biases. After each feeding session, cages bearing experimental insects were returned to new positions on the shelves to prevent position bias. Parameters measured during this study included nutrition composition of formulated feeds; developmental time of *G. madagascarensis* juveniles per feed; surviving insects; body mass; body length; quantity of feed consumed; feed conversion ratio; cost of feeding per kg live mass gain; nutrient composition of the crickets fed on the formulated feed and reference and cost of feed production of one kilogram of feed. These parameters are explained in respective subheadings below.

### 2.5. Nutritional Analysis of the Nine Formulated Feeds and the Reference Feed

Before the start of the experiment, the nutritional profiles of the nine formulated feeds and reference feed (chicken feed) were analyzed by the Nutrition Laboratory of the International Livestock Research Institute, Nairobi, Kenya. Crude fat (%), crude protein (%), crude fiber (%), and ash (%) contents of the formulated feeds and the reference were analyzed following the official methods of the Association of Official Analytic Chemists (AOAC) [24]. The percentage of moisture in each formulated feed and reference was determined by drying each feed sample in an oven set at 110 °C for 24 h. The dried samples were cooled to room temperature in a desiccator before reweighing using a Mettler P1210 analytical balance to attain a constant weight. The carbohydrate content was calculated by subtracting the percentage content of crude protein, crude fat, crude fiber, and ash from 100% of dry matter [25]. The gross energy value (kcal/kg) was calculated by summing the percentages of proteins and carbohydrates and multiplying the total by forty before adding it to the total lipids multiplied by a factor of ninety [26]. An atomic absorption spectrometer (AAS) was used to determine the mineral composition of each formulated feed [27].

**Table 2:** Nutrition content of experimental feeds (dry matter basis).

Nutrition Composition	Feed									
	CFA	CFB	CFC	CFD	CFE	CFF	CFG	CFH	CFI	CFJ
Iron (Fe) (mg/kg)	228.1	61.1	171.1	327.2	288.2	212.5	881.4	172.6	46.2	38.4
Copper (Cu) (mg/kg)	16.2	5.3	6.8	18.0	7.3	19.7	9.7	8.6	5.37	3.5
Zinc (Zn) (mg/kg)	67.0	49.5	37.1	62.5	45.0	28.8	64.2	35.8	57.9	18.9
Manganese (Mn) (mg/kg)	63.9	73.9	35.9	52.8	57.6	10.2	79.3	52.8	83.6	85.2
Sodium (Na) (mg/kg)	1065.4	100.0	1939.3	1935.0	2033.4	1040.8	2399.9	1935.0	21.3	19.3
Magnesium (Mg) (%)	0.2	0.01	0.2	0.3	0.2	0.2	0.3	0.2	0.3	0.3
Calcium (Ca) (%)	0.5	0.01	0.9	0.9	0.7	0.1	1.1	0.6	0.1	0.1
Phosphorus (P) (%)	0.2	0.02	0.2	0.2	0.3	0.2	0.2	0.2	0.3	0.02
Potassium (K) (%)	1.3	1.3	1.2	1.7	1.3	1.2	1.6	1.0	1.2	1.5
Moisture (%)	9.8	9.5	12.9	7.2	11.6	9.5	7.9	8.8	9.9	12.0
Crude protein (%)	28.0	25.0	24.5	24.0	23.5	22.5	21.5	20.0	14.5	13.5
Crude fat (%)	9.3	3.4	3.8	7.1	2.1	1.9	6.3	5.3	3.4	1.3
Crude ash (%)	6.3	5.3	8.4	10.1	10.1	6.4	11.8	11.3	5.3	5.2
Crude fiber (%)	2.5	10.0	22.7	8.8	10.6	6.2	12.5	12.8	9.98	11.4
Available carbohydrates (%)	44.1	46.8	27.7	42.8	42.1	53.5	40.0	41.8	56.9	56.6
Energy (Kcal/kg)	3721.0	3178.0	2430.0	3311.0	2813.0	3211.0	3027.0	2949.0	3162.0	2921.0

Note: CFA—16% soybean meal + 22% a mixture of fine wheat and wheat flour + 15% broken rice+ 25% maize + 5% bergafats + 1% lysine amino acid +1% DCP + 1% methionine + 1% vitamin-mineral premix + 1% lime + 15% fish meal; CFB—98% cassava leaves + 1% sugar + 1% baking powder; CFC—20% silver leaf desmodium + 20% wheat bran + 40% cowpea bran + 20% maize bran; CFD—30% tropical African morning glory, 30% maize bran, 20% navy bean bran, 10% maize germ, 10% rice bran; CFE—20% tropical African morning glory, CFF—30% tropical African morning glory + 40% cassava leaves powder + 20% cowpea bran + 10% of navy bean bran; CFG—20% tropical African morning glory, 20% gallant soldier, 15% cassava leaves, 20% cowpea bran, 10% navy bean bran, 10% maize bran, 5% wheat bran; CFH—30% cassava leaves + gallant soldier + 20% cowpea bran + 10% tropical African morning glory + 10% taro leaves; CFI—99% wheat bran and 1% baking powder; CFJ—33% maize bran, 33% cassava tuber bran, 33% wheat bran, 1% baking powder; DCP is dicalcium phosphate.

### 2.6. Effect of Formulated Feeds on the Development and Survival Rate of Gryllus madagascarensis

To determine the influence of the nine formulated feeds and the reference feed on the development time and survivorship of *G. madagascarensis*, 150 one-day-old juveniles were introduced individually into ventilated plastic containers, forming a group to be provided one type of formulated feed. The same procedure was repeated for all nine formulated and reference feeds. An individual juvenile, therefore, was treated as a replicate (*n* = 150 per feed). At the start of the experiment, each juvenile was given 0.2 g of feed on a plastic Petri dish measuring 4 cm in diameter; as the crickets increased in size, the feed was adjusted to 0.5 g. Each juvenile was provided water ad libitum and can in soaked cotton balls measuring 3 cm in diameter. Then, every juvenile was offered a piece of egg crate (5 cm by 5 cm by 5 cm) for hiding. The feeds and cotton balls for water in all cages were replaced after three days to prevent the development of disease pathogens that could kill the crickets. Before cleaning and replacing feeds and cotton balls for water, each cage possessing a cricket was moved into a larger box. The cricket hiding in a piece of egg crate was transferred into another small plastic container inside the large box. This was performed to prevent the cricket from escaping. After each cage was cleaned and supplied with fresh feed and water in a cotton ball, the cricket was returned to the cage to continue to feed. The cages were returned to the shelves in different positions to avoid position biases. Unconsumed feed and cricket droppings from each cage were put into labeled Petri dishes awaiting separation.

To separate the cricket droppings from the unconsumed feed, the mixture from each cage was transferred into one end of the separating plastic box and spread out using a camel hairbrush. The box was then slightly tilted to create a gradient for the large cylindrical cricket droppings to separate from the mixture and roll down to the lower end of the separating box. The unconsumed feed consisting of smaller particles remained at the upper end of the separating box. To help the cricket droppings roll away from the mixture, the box was gently tapped using a finger on the underside at the point where the mixture was placed. The gentle tapping of the box was performed several times until all the droppings were separated from the uneaten feed. If some feed rolled down with the droppings, it was separated using the camel hairbrush. The separated droppings and unconsumed feed were transferred into labeled glass Petri dishes. Then, the separated unconsumed feed and droppings were transferred and dried in a 70 °C oven for four hours. After drying, the unconsumed feeds and respective cricket droppings were weighed, and their masses were recorded.

*Gryllus madagascarensis* juveniles in the cages were monitored every six hours daily as they developed until the adult stage. Any mortality that occurred was recorded. Nymph development time was calculated as the number of days between the hatching of the egg and the time of the emergence of the adult cricket [2]. The percent survival rate was obtained by determining the number of individuals alive at the end of the experiment divided by the initial number multiplied by 100 [20].

### 2.7. Effect of Formulated Feeds and Reference Feed on Wet Body Mass and Body Length of G. madagascarensis

To determine the wet body mass and body length of male and female *G. madagascarensis*, 25 male and 25 female crickets (*n* = 50) were randomly picked from the plastic containers 24 h after emergence to the adult stage from the last juvenile fed on each formulated feed and introduced individually into a transparent plastic cup (7.5 cm diameter × 12.5 cm height) covered with a transparent Petri dish to prevent the cricket from jumping out. The cricket in the cup was allowed 3–5 min to settle down, then its length was measured by placing a ruler under the container parallel to the cricket. The lower limit of measurement by the ruler was 1 mm. The wet body mass of the cricket was also recorded using a digital electronic weighing machine with 0.0001 g sensitivity (Kern and Sohn, Ballngen, Germany). The cricket in the cup hardly moved when weighed. The body mass of each cricket was recorded when the reading on the balance screen stopped changing. The body mass of the cricket was recorded as the total mass of the cup containing the cricket minus the mass of the transparent cup [2]. This method of measuring cricket body mass and body size is the most suitable because it eliminates touching the crickets by hand. This helps ensure that delicate body parts such as the hind limbs and antennae do not break off from the cricket. Further, this avoids exposing the crickets to disease-causing pathogens from contaminated hands. The sex of the crickets was determined by checking the presence of an ovipositor in females [28].

### 2.8. Effect of Formulated Feeds and Reference Feed on Daily Feed Intake, Average Feed Conversion Ratio (FCR), and Cost of Feeding per kg Live Weight Gain of G. madagascarensis

To assess the impact of the formulated feed and the reference on feed conversion ratio (FCR), 1500 nymphs were divided into 10 groups (150 nymphs per group) and kept individually in cages. The nymphs were randomly introduced to the nine formulated feeds and the reference feed. Each cricket was given more feed than it could finish (0.2 g of each feed treatment) and water. All 150 individual crickets in each group were weighed separately every seven days, as reported by [14]. The amount of formulated and reference feed consumed by an individual cricket was weighed when the feed was exchanged after three days until the time of adult emergence. Daily feed intake (kg) was calculated by recording the quantity of formulated or reference feeds consumed (g) each week and dividing it by seven days. The average daily mass gain was obtained by subtracting the initial body mass of the cricket from the final body mass gained since the last weighing divided by the time required for the nymphs to reach the adult stage. The FCR was determined by dividing the daily feed intake by the average mass gained by the cricket. Formulated feeds with optimum protein content for the crickets have low FCR compared with feeds with high and low protein content. Therefore, the low FCR demonstrates increased feed efficiency, leading to reduced cost of cricket production. By comparison, feeds with high FCR result in decreased feed efficiency, leading to increased production costs that make the final cricket food product expensive. The number of crickets needed to produce one kilogram of live weight gain per formulated feed and reference feed was calculated by dividing 1000 g, equivalent to one kilogram of crickets, by the final mass gained per cricket. The consumed amount of feed (kgDM) per kg live weight gain was determined by multiplying the number of crickets making one kilogram per feed with the feed consumed by each cricket to the adult stage and dividing by 1000. The cost of feeding crickets in each formulated feed and reference feed was calculated as the cost of feeding the crickets per kg live weight gain = each formulated feed or reference feed cost per kg × food consumed by one kilogram of crickets per feed. The mean cost of feeding per kg live weight gain for each formulated feed or reference feed was obtained by multiplying the FCR obtained from the mean of the 150 replicates × feed cost per kg. The average cost of feeding per kg live weight gain was then calculated. The first feeding experiment was conducted from April to June 2023, the second feeding experiment from July to September 2023, and the last feeding experiment from January 2024 to March 2024. We split the feeding program into three periods to assess seasonal/climate differences.

### 2.9. Effect of Formulated Feeds on the Reproductive Performance of Gryllus madagascarensis

To assess the effects of nine formulated feeds and the reference feed on reproductive parameters, 10 males and 10 females (10 pairs) (*n* = 20) of newly molted adult *G. madagascarensis* were subjected to each of the formulated feeds and reference feed and followed daily until they died. The 100 pairs used for the experiment were easily obtained since the eggs to carry out the test experiments were collected within two-hour periods. This meant the eggs hatched at the same time, leading to adult crickets also emerging at the same time. A male and female cricket were introduced into a well-ventilated transparent plastic cage measuring 22 × 16 × 15 cm (1200 mL Rectangle Super 2; Aristo Manufacturers Limited, India), and the cages were put randomly on shelves in the experiment room. The paired crickets were maintained under conditions similar to those mentioned for the feeding regimes. Each cage with male and female crickets was offered a moist cotton ball (70% moist), which served as an oviposition medium, at 6 p.m. daily. The female crickets were allowed to lay eggs for 24 h. The cotton balls were then withdrawn after 24 h and replaced with a fresh one. Before replacing the cotton balls, the cages were cleaned by removing the cricket droppings. The fresh feed and cotton balls were introduced into the cages; the position of each container was changed randomly to avoid the possibility of location influence [28]. The cotton ball for oviposition was replaced daily throughout the lifetime of the crickets. The presence of eggs on withdrawn cotton balls was checked by opening the balls and separating them into thin cotton sheets. The cotton balls did not have eggs in the first few days. The timespan when no eggs were recorded in the cotton balls was the preoviposition period. Preoviposition time was obtained from the emergence time of the adult female cricket from the last juvenile stage to the first day of egg laying [2]. The preoviposition period varied with the different formulated feeds and the reference feed fed to the pair of crickets. Any eggs laid by the female crickets could be seen deposited in the cotton balls. The trapped eggs from each gravid female were counted and incubated in 1000 mL rectangular plastic cages measuring 16 × 10 × 7.5 cm (Super 1; Aristoplast Products Pvt. Ltd, Mumbai, India), ventilated with 0.2 mm gauge wire mesh on the lid at the top. The eggs were put on a wet sheet of cotton wool and then covered with another sheet of cotton. The cotton wool was sprinkled with water every morning to prevent the eggs from drying out. The egg containers were checked after six hours daily for newly hatched nymphs. Nymph numbers were recorded until hatching stopped completely. Fecundity was calculated as the sum of the number of eggs deposited by each female in a lifetime [2]. Egg incubation was determined from the day the egg was laid until hatching occurred [28]. During observation of the progress of egg hatching, eggshells were counted and removed with a fine brush [19]. Percent egg hatchability was calculated as the number of hatched eggs divided by the total number of eggs oviposited by each female cricket multiplied by 100 [29]. The time the eggs take to hatch was calculated from the onset of egg hatch to the time the eggs stopped hatching completely. The determination of the right feed with optimum nutrients to reduce the preoviposition period, increase fecundity, reduce the time of egg hatching, and increase the fertility of the eggs in *G. madagascarensis* is, therefore, an integral requirement for increasing yield, profits, and sustainable farming of the cricket.

### 2.10. Nutritional Analysis of Gryllus madagascarensis Fed on Nine Formulated Feeds and the Reference Feed

#### 2.10.1. Cricket Samples and Their Preparation

An equal number of male and female adults of *G. madagascarensis* on nine formulated diets and a reference feed were harvested separately into Ziploc plastic bags. The harvested crickets were snap-frozen, washed in clean tap water to remove dirt, and then frozen again at −20 °C to kill them [30]. The dead crickets were stored at −20 °C to await proximate and mineral elements and fatty acid extraction. To prepare the crickets for proximate and mineral elements and fatty acid extraction and analyses, the frozen cricket samples were removed from the freezer and allowed to thaw at room temperature before being dried in a convection oven (Memmert, Germany) set at 40 °C for 12 h. The samples were milled into a fine powder using a blender (MIKA MBLR2999WB manufactured by SINREEN, China). The powders from the cricket samples were then stored in air and watertight dark bottles to prevent light from initiating their oxidation. Samples not used immediately for proximate and mineral elements and fatty acid analyses were stored in a freezer at −20 °C to prevent spoilage and other chemical reactions due to high temperatures. 

#### 2.10.2. Proximate Analysis

The proximate composition of *G. madagascarensis* fed on nine formulated feeds and the reference feed samples were analyzed in triplicate following the Association of Official Analytical Chemists [24] methods at the Nutrition Laboratory of International Livestock Research Institute, Nairobi, Kenya. Before the crickets were harvested for nutritional analyses, they were starved for twenty-four hours to ensure their guts were cleared of feed by the processes of digestion, absorption, and egestion of frass. This was to prevent undigested feed in the gut from affecting the results. Crickets from each feed trial were harvested after attaining the adult stage and killed separately by blanching in boiling water for one minute before being dried in an oven set at 40 °C with convection (Memmert, Germany) for 12 h, milled, and analyzed for proximate analysis. The amount of moisture was determined by drying the sample in a 135 °C oven for 2 h (Method No. 930.15) [24]. Ash content was obtained by combusting samples at 550 °C in a muffle furnace until a constant weight was achieved (Method No. 930.05) [24]. The crude protein composition of both the feeds and cricket powder was determined by following the automatic Kjeldahl method (Method No. 2001.11), and the obtained values were multiplied by a nitrogen factor of 5.25 [31]. The total fat content was determined following the Soxhlet method (Method No. 2003.05) [24]. The crude fiber was determined by ignition on the weight loss of residue after hydrolysis with acid and alkali solutions (Method number 978.10) [23]. Available carbohydrate content was obtained by difference (100% DM—(% moisture + % crude protein + % crude lipid + % crude ash + crude fiber)) [25]. The gross energy (kcal/kg) supplied by the various cricket samples fed different feeds was obtained by summing the percentages of proteins and carbohydrates and multiplying the total by 40 before adding it to the total lipids multiplied by a factor of 90 [32].

#### 2.10.3. Mineral Analysis

Both macro- and micromineral analyses of the formulated feeds, chicken feed, and cricket powders were conducted using a standard method [32] at the Nutrition Laboratory of the International Livestock Research Institute, Nairobi, Kenya. To 0.5 g of each sample, eight mL of concentrated nitric acid (16.2 mol/L) (VWR Chemicals, Fontenaysous-Bois, France) and 2 mL of 9.8 mol/L hydrogen peroxide (Sigma-Aldrich, USA) were added and left overnight in a fume chamber to digest. The digested samples were then subjected to further digestion at 75 °C for 30 min, 120 °C for 20 min, 180 °C for 20 min, and 200 °C for 10 min in a temperature-controlled block digester (Model TE007-A, TECNAL, São Paulo, SP, Brazil). The resulting wet ashes were cooled in a fume hood before being dissolved in 0.4 mol/L nitric acid and diluted ten-fold to appropriate concentrations based on the mineral element and the resulting calibration curve. The calibrated curves for quantifying each mineral element in each sample were determined by measuring standard solutions from certified stock solutions (Chem Lab, Zedelgem, Belgium) and diluted to give calibration standards of 400, 800, 2000, and 4000 g/L. The quantity of each assessed micro- or macroelement and the standard were obtained from an inductively coupled plasma–optical emission spectrometry (ICP-OES) (Optima 4300™ DV ICP-OES, Perkin Elmer, Wellesley MA, USA). To calibrate the external standards and record the data, Perkin Elmer Winlab 32 software (Perkin Elmer, USA) was used [33,34]. All samples were analyzed in triplicate.

#### 2.10.4. Fatty Acid Analysis

Gas chromatography–spectrometry was used to analyze the fatty acids profile in the feed ingredients and cricket samples fed experimental feeds at the Nutrition Laboratory of International Livestock Research Institute, Nairobi, Kenya. To extract oil from each sample, a mixed ratio of 20 mL dichloromethane liquid to methanol liquid (2:1 *v*/*v*) was added to 10 g of each sample [35]. About 100 mg of oils from each was esterified by adding 1 mL of sodium methoxide solution (15 mg/mL) and then vortexing the mixtures for a minute. After vortexing, the mixtures were sonicated for 10 min and incubated for one hour in a water bath set at 70 °C. Then, 100 mL of distilled deionized water was added to decrease the reaction before vortexing for another minute. To the extracted oils, one milliliter of gas chromatography (GC)-grade hexane (Sigma-Aldrich, St. Louis, MO, USA) was added, forming fatty acids methyl esters (FAMEs). The FAMEs were centrifuged at 14,000 rpm for 20 min to develop the supernatant. Each supernatant collected was passed through anhydrous sodium sulfate to dry before being filtered. One microliter of each dry supernatant was analyzed by GC–MS on a 7890A gas chromatograph attached to a 5975C mass selective detector (Agilent Technologies Inc., Santa Clara, CA, USA). The GC used comprised a (5%-phenyl)-methylpolysiloxane (HP5 MS) low-bleed capillary column (30 m × 0.25 mm i.d., 0.25 μm; J and W, Folsom, CA, USA). Helium was used as a transporting gas at 1.25 mL/min flow rate. At an increasing rate of 10 °C/min, the oven temperature was programmed from 35 °C to 285 °C, with the starting and stopping temperatures kept at 5 min and 20.4 min, respectively. The ion source and the quadrupole mass selective detector temperatures were kept constant at 230 °C and 180 °C, respectively. The spectrum masses from electron impact (EI) were acquired at an acceleration energy of 70 eV, and the fragment ions were analyzed over the 40–550 m/z mass range in the full-scan mode, with the filament delay time set at 3.3 min. Octadecanoic acid (≥95% purity) (Sigma-Aldrich, St. Louis, MO, USA) was used to prepare serial dilutions of authentic standard methyl octadecanoate (0.2–125 ng/μL), which were also analyzed by GC–MS in full-scan mode to generate a linear calibration curve (peak area vs. concentration) with the following equation: Y = [5 × 10^7^x] + [2 × 10^7^]; R2 = 0.9997, and used for external quantification of the various fatty acids. ChemStation B.02.02 software was used for the data acquisition, and the compounds were identified by comparison of mass spectral data and retention times with those of authentic standards and reference spectra published by the library–MS databases: National Institute of Standards and Technology (NIST) 05, 08, and 11. Determination of the FAMEs was performed in triplicate.

### 2.11. Cost of the Experimental Feeds

The price of each cricket feed developed was calculated by totaling the price of each ingredient required to prepare one kilogram of each feed. Prices of ingredients were those prevailing in the retail market in Antananarivo in 2024. Given that feeds were blended from selected weeds and agro-byproducts that were locally available in large quantities and cheap, they have great potential to be used as an alternative feed source for crickets, capable of replacing expensive chicken feed in the large-scale farming of crickets in Madagascar.

### 2.12. Statistical Analysis

All data were analyzed using R version 4.3.1 [36] statistical software. Results are expressed as mean ± standard deviation in the case of nutrition analysis and mean ± standard error (SE) for developmental time, surviving insects, body mass, body length, quantity of feed consumed, feed conversion ratio, cost of feeding per kg live mass gain, preoviposition period, number of eggs, incubation period and egg hatchability. Data were subjected to a one-way analysis of variance (ANOVA). Post hoc multiple comparison, the Student–Newman–Keul (SNK) test was conducted to determine significant differences in means of developmental time, surviving insects, body mass, body length, quantity of feed consumed, feed conversion ratio, cost of feeding per kg live mass gain, nutrient composition, and cost of feed production among dietary treatments that were normally distributed and had the same variance. Before running the ANOVA test, the normality (Shapiro test: *p* > 0.05) and homoscedasticity (Bartlett test: *p* > 0.05) assumptions were checked. The threshold of statistical significance was set at *p* < 0.05.

## 3. Results

### 3.1. Proximate and Mineral Elements Content of Formulated Feeds and Reference Feed

The proximate content of nine formulated feeds and reference feed per 100 g dry weight are shown in Table 2. The range of energy supplied by the formulated feed was between 324.0 and 356.3 kcal/100 g compared with the dry weight of the reference feed (381.9 kcal/100 g). The major component in all samples was carbohydrates (46.2–68.1 g/100 g dry weight), with the distribution of dietary fiber ranging from 2.5 to 22.7 g/100 g dry weight. Protein and fat contents varied between 13.5 and 28.0% and 1.3 and 9.3 g/100 g dry weight, respectively, and ash content ranged from 5.2 to 11.8 g/100 g dry weight. Feed CFA had the highest crude protein (28.0%) and crude fat (9.3%) content, while feed CFI had the highest carbohydrate (56.9%) content and CFC the least (27.7%). Feed CFC had the largest quantity of fiber (22.7%), while the smallest amount was recorded in CFF (6.2%). A higher ash content was recorded in feed CFG (11.8%) than in other feeds. CFG feed contained the highest quantity of minerals: iron (881.4 mg/kg), zinc (64.2 mg/kg), manganese (79.3 mg/kg), and sodium (2399.9 mg/kg). Feed CFJ had the lowest quantity of iron (38.4 mg/kg), copper (3.5 mg/kg), zinc (18.9 mg/kg), and sodium (19.3 mg/kg). All feeds contained more calcium than magnesium. Most feeds, except CFB and CFJ, contained phosphorus ranging between 0.2 and 0.3 mg/kg (Table 2).

### 3.2. Effect of Formulated Feeds and Reference Feed on the Development Time of G. madagascarensis

**Figure 1:**
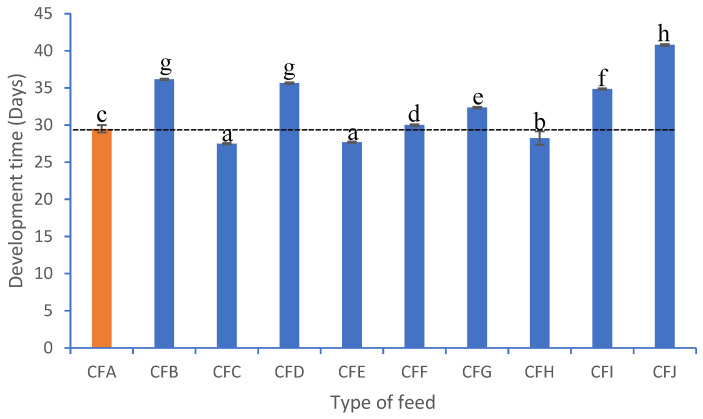
The mean values ± standard deviation of development time of *Gryllus madagascarensis* cricket reared on different formulated and reference feeds. Different letters in a column indicate significant differences, Student–Newman–Keul test (*p* < 0.05). CFA—16% soybean meal + 22% a mixture of fine wheat and wheat flour + 15% broken rice+ 25% maize + 5% bergafats + 1% lysine amino acid +1% DCP + 1% methionine + 1% vitamin-mineral premix + 1% lime + 15% fish meal; CFB—98% cassava leaves + 1% sugar + 1% baking powder; CFC—20% silver leaf desmodium + 20% wheat bran + 40% cowpea bran + 20% maize bran; CFD—30% tropical African morning glory, 30% maize bran, 20% navy bean bran, 10% maize germ, 10% rice bran; CFE—20% tropical African morning glory, CFF—30% tropical African morning glory + 40% cassava leaves powder + 20% cowpea bran + 10% of navy bean bran; CFG—20% tropical African morning glory, 20% gallant soldier, 15% cassava leaves, 20% cowpea bran, 10% navy bean bran, 10% maize bran, 5% wheat bran; CFH—30% cassava leaves + gallant soldier + 20% cowpea bran + 10% tropical African morning glory + 10% taro leaves; CFI—99% wheat bran and 1% baking powder; CFJ—33% maize bran, 33% cassava tuber bran, 33% wheat bran, 1% baking powder; DCP is dicalcium phosphate.

The development time for farmed crickets is an important criterion for evaluating feed performance on cricket yields. The time taken by juveniles to develop into adults differed significantly across the formulated and reference feeds (F_9, 1490_ = 553.2, *p* < 0.001) (Figure 1). The shortest average development period, 27.5 ± 0.2 days, was recorded with feed CFC compared with the reference feed (29.3 ± 0.2) days. The longest developmental time was associated with CFJ feed (40.8 ± 0.2) days.

### 3.3. Effect of Formulated and Reference Feed on Wet Body Mass, Body Size, Feed Conversion Ratio, Survivorship, and Cost of Producing One Kilogram of G. madagascarensis

**Figure 2:**
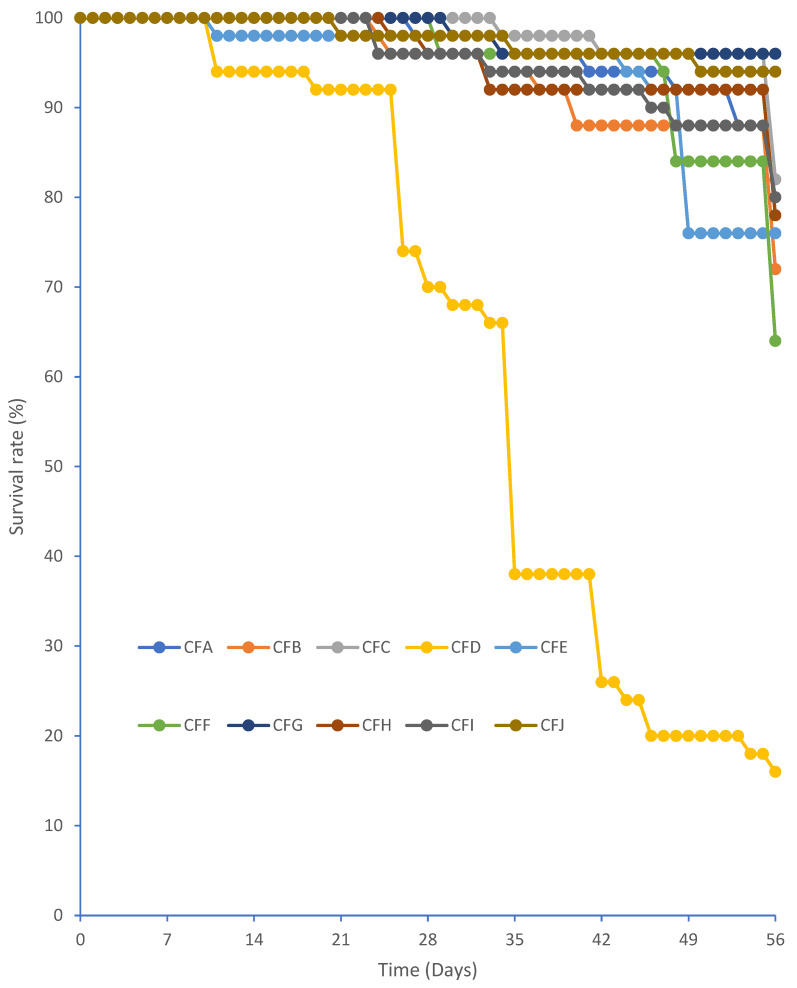
Survival rate of *Gryllus madagascarensis* reared on different feeds: CFA—16% soybean meal + 22% a mixture of fine wheat and wheat flour + 15% broken rice + 25% maize + 5% bergafats + 1% lysine amino acid + 1% DCP + 1% methionine + 1% vitamin-mineral premix + 1% lime + 15% fish meal; CFB—98% cassava leaves + 1% sugar + 1% baking powder; CFC—20% silver leaf desmodium + 20% wheat bran + 40% cowpea bran + 20% maize bran; CFD—30% tropical African morning glory, 30% maize bran, 20% navy bean bran, 10% maize germ, 10% rice bran; CFE—20% tropical African morning glory, CFF—30% tropical African morning glory + 40% cassava leaves powder + 20% cowpea bran + 10% of navy bean bran; CFG—20% tropical African morning glory, 20% gallant soldier, 15% cassava leaves, 20% cowpea bran, 10% navy bean bran, 10% maize bran, 5% wheat bran; CFH—30% cassava leaves + gallant soldier + 20% cowpea bran + 10% tropical African morning glory + 10% taro leaves; CFI—99% wheat bran and 1% baking powder; CFJ—33% maize bran, 33% cassava tuber bran, 33% wheat bran, 1% baking powder; DCP is dicalcium phosphate.

Feed influences farming output by impacting cricket mass and size. In the current study, the body mass and size of the cricket were measured to determine the most suitable formulated feed (Table 3). The mean mass of all newly hatched *G. madagascarensis* nymphs was 0.001 ± 0.000 g; the mean body length at the beginning of the feeding experiment was 2.0 ± 0.0 mm (Table 3). Crickets fed on CFG, CFE, and CFC had better growth rates than crickets fed on the other formulated feeds and the reference feed. Adult cricket body mass differed significantly among crickets fed on different formulated and reference feeds (F_9, 490_ = 14,693, *p* < 0.001). Crickets fed CFG had the highest adult mean body mass (1.15 g) compared with individuals fed the reference feed (0.81 g), while those fed feed CFJ had the lowest mean body mass (0.20 g) (Table 3). Similar observations were made for the body size of adult crickets raised on various formulated feeds and the reference feed (F_9, 490_ = 1320.00, *p* < 0.001) (Table 3). Daily feed intake per cricket raised on CFG (0.053 gDM), which resulted in the heaviest crickets, was comparable to that of the reference feeds (0.050 gDM) (Table 3). The highest daily intake was recorded on crickets provided with CFI (0.090 gDM), while the lowest daily intake was recorded on CFJ (0.0163 g). On the other hand, crickets fed CFC showed the highest average daily weight gain (0.038 g), followed by crickets fed CFG (0.036 g) and CFE (0.035 g). The lowest average daily weight gain (0.005 g) was among crickets raised on CFJ. Crickets fed on formulated and reference feeds exhibited a highly significant feed conversion ratio (FCR) (F_9, 1490_ = 13,008, *p* < 0.001). The groups provided with feeds CFG, CFE, and CFC had the lowest FCR (1.7) compared with the reference feed (1.8) and other formulated feeds. The feed used to produce one kilogram of live crickets varied significantly across the feeds (F_9,1490_ = 6490, *p* < 0.001). The crickets fed CFG and CFE consumed the least amount of feed (0.31 and 0.32 kgDM, respectively), while crickets fed on CFJ consumed the largest quantity of feed (1.40 kgDM). Crickets provided CFG, CFE, and CFC had the highest survivorship (98% each) compared with reference feed (Figure 2). Crickets reared on CFD had the lowest survivorship (66%) (Figure 2). The cost of feeding per kg live mass gain of crickets (in Malagasy ariary) differed significantly by feed (F_9,1490_ = 357,489, *p* < 0.001). The cheapest mean cost per kg live mass was observed in crickets fed using CFH (216 Malagasy ariary) and CFG (217 Malagasy ariary), while the most expensive mean cost per kg live mass was recorded in crickets fed feed CFJ (1507 Malagasy ariary) and chicken feed (1444 Malagasy ariary). Crickets reared on CFG resulted in the lowest number of crickets, making up one kilogram of live weight, while crickets fed on CFJ resulted in the highest number of crickets required to make one kilogram of live weight.

**Table 3:** *Gryllus madagascarensis* mean body mass gain, body length, feed conversion ratio (FCR), quantity of feed consumed per kg live weight gain, and cost of feeding per kg live mass gain when fed on nine formulated feeds and the reference feed.

Feed	Initial Mean Body Length (mm)	Final Mean Body Length (mm)	Initial Mean Body Mass (g)	Final Mean Body Mass Gain (g)	Daily Feed Intake per Cricket (gDM)	Average Daily Gain (g)	FCR	Consumed Amount of Feed (kgDM) per kg Live Weight Gain	Cost of Feeding per kg Live Weight Gain (Malagasy Ariary)
CFA	2.00 ± 0.00	23.55 ± 0.08 g	0.001 ± 0.00	0.81 ± 0.01 g	0.050 ± 0.001 b	0.027 ± 0.001 g	1.85 ± 0.01 c	0.38 ± 0.01 ef	1444.00 ± 0.83 h
CFB	2.00 ± 0.00	24.06 ± 0.05 f	0.001 ± 0.00	0.78 ± 0.01 h	0.070 ± 0.001 h	0.022 ± 0.001 h	3.18 ± 0.01 f	0.37 ± 0.01 de	383.76 ± 0.18 f
CFC	2.00 ± 0.00	25.86 ± 0.15 b	0.001 ± 0.00	1.05 ± 0.01 c	0.065 ± 0.001 g	0.038 ± 0.001 a	1.71 ± 0.01 a	0.34 ± 0.01 b	272.00 ± 0.52 c
CFD	2.00 ± 0.00	25.59 ± 0.08 c	0.001 ± 0.00	1.03 ± 0.01 d	0.058 ± 0.001 d	0.029 ± 0.001 e	1.93 ± 0.01 d	0.34 ± 0.01 b	306.00 ± 0.42 d
CFE	2.00 ± 0.00	26.69 ± 0.09 a	0.001 ± 0.00	1.11 ± 0.01 b	0.060 ± 0.001 e	0.035 ± 0.001 c	1.71 ± 0.01 a	0.32 ± 0.01 a	320.00 ± 2.11 e
CFF	2.00 ± 0.00	25.07 ± 0.05 d	0.001 ± 0.00	0.94 ± 0.01 e	0.060 ± 0.001 f	0.031 ± 0.001 d	1.94 ± 0.01 e	0.35 ± 0.01 c	245.00 ± 1.02 b
CFG	2.00 ± 0.00	26.80 ± 0.09 a	0.001 ± 0.00	1.15 ± 0.01 a	0.053 ± 0.001 c	0.036 ± 0.001 b	1.71 ± 0.01 a	0.31 ± 0.01 a	217.00 ± 0.30 a
CFH	2.00 ± 0.00	24.43 ± 0.06 e	0.001 ± 0.00	0.85 ± 0.01 f	0.056 ± 0.001 d	0.030 ± 0.001 f	1.83 ± 0.01 b	0.36 ± 0.01 cd	216.00 ± 0.37 a
CFI	2.00 ± 0.00	22.09 ± 0.09 h	0.001 ± 0.00	0.69 ± 0.01 i	0.090 ± 0.001 i	0.020 ± 0.001 i	2.25 ± 0.01 e	0.41 ± 0.01 f	592.04 ± 0.31 g
CFJ	2.00 ± 0.00	13.50 ± 0.11 i	0.001 ± 0.00	0.20 ± 0.01 j	0.0163 ± 0.001 a	0.005 ± 0.001 j	3.26 ± 0.01 g	1.40 ± 0.01 g	1507.10 ± 0.14 i

The results are expressed as mean values ± standard deviation of three determinations on a dry matter (DM) basis. Different letters in a column indicate significant differences, as determined by Student–Newman–Keul test (*p* < 0.05): CFA—16% soybean meal + 22% a mixture of fine wheat and wheat flour + 15% broken rice+ 25% maize + 5% bergafats + 1% lysine amino acid +1% DCP + 1% methionine + 1% vitamin-mineral premix + 1% lime + 15% fish meal; CFB—98% cassava leaves + 1% sugar + 1% baking powder; CFC—20% silver leaf desmodium + 20% wheat bran + 40% cowpea bran + 20% maize bran; CFD—30% tropical African morning glory, 30% maize bran, 20% navy bean bran, 10% maize germ, 10% rice bran; CFE—20% tropical African morning glory, CFF—30% tropical African morning glory + 40% cassava leaves powder + 20% cowpea bran + 10% of navy bean bran; CFG—20% tropical African morning glory, 20% gallant soldier, 15% cassava leaves, 20% cowpea bran, 10% navy bean bran, 10% maize bran, 5% wheat bran; CFH—30% cassava leaves + gallant soldier + 20% cowpea bran + 10% tropical African morning glory + 10% taro leaves; CFI—99% wheat bran and 1% baking powder; CFJ—33% maize bran, 33% cassava tuber bran, 33% wheat bran, 1% baking powder; DCP is dicalcium phosphate.

### 3.4. Effect of Formulated and Reference Feeds on the Reproductive Parameters of G. madagascarensis

Reproductive efficiency and success are essential when establishing and expanding a cricket colony. Therefore, we assessed the influence of formulated feeds on four major reproductive characteristics, such as the duration of preoviposition (time taken by an adult female to start ovipositing eggs), female fecundity, egg hatchability, and incubation time. The duration of preoviposition varied significantly with different single-plant products and blended reference feed (F_9,90_ = 8.12, *p* < 0.001). The preoviposition time was shortest in females raised on CFG (~2 days), and the longest duration was among those fed CFB (~4 days) (Table 4). Fecundity was also significantly impacted by the formulated feeds and reference feed (F_9,90_ = 39.03, *p* < 0.001; Table 4). Crickets fed CFG produced the highest number of eggs, while crickets raised on CFJ laid the least number of eggs.

The shortest incubation time was recorded in females fed with CFG, (6.6 days), a shorter duration than with the reference feed (7 days), while the longest was observed in females raised on CFJ (~10 days) (Table 4). The different formulated feeds tested had a strong effect on egg hatchability (F_9,90_ = 1320, *p* < 0.001). The highest hatchability was recorded for eggs laid by females fed with CFG (93.6%) compared with the reference feed (92.4%), while the lowest hatchability was recorded for eggs from females raised on CFJ (67.5% (Table 4). The feed used to grow the females also significantly impacted the incubation time of eggs (F_9,90_ = 21.48, *p* < 0.001).

**Table 4:** The effects of different formulated and reference feeds on the reproductive parameters of *G. madagascariensis*.

Formulated Feeds and Blended Reference Feed	Preoviposition Time (Days)	Eggs	Egg incubation Time (Days)	Egg Hatchability (%)
CFA	2.0 ± 0.08 bc	2329.50 ± 20.06 abc	7.10 ± 0.28 c	92.40 ± 0.22 bc
CFB	3.80 ± 0.04 c	1944.80 ± 50.74 d	8.60 ± 0.22 c	88.30 ± 0.31 e
CFC	1.80 ± 0.06 ab	2424.40 ± 67.83 ab	7.00 ± 0.30 c	93.50 ± 0.17 a
CFD	1.80 ± 0.05 ab	2375.80 ± 41.02 abc	6.90 ± 0.18 b	92.8 ± 0.29 ab
CFE	2.60 ± 0.08 bc	2505.70 ± 46.23 a	6.70 ± 0.21 b	93.10 ± 0.10 ab
CFF	2.00 ± 0.07 bc	2151.40 ± 39.65 cd	8.20 ± 0.13 c	86.60 ± 0.16 d
CFG	1.70 ± 0.07 a	2554.10 ± 12.78 a	6.60 ± 0.16 a	93.60 ± 16 a
CFH	2.70 ± 0.09 c	2233.70 ± 64.19 bc	7.30 ± 0.15 c	88.6 ± 0.16 e
CFI	3.20 ± 0.10 c	1967.10 ± 53.95 d	7.10 ± 0.10 c	91.90 ± 0.10 c
CFJ	3.10 ± 0.10 c	1130.60 ± 56.48 e	9.50 ± 0.22 c	67.50 ± 0.34 f

The results are expressed as mean values ± standard deviation of three determinations on a dry matter (DM) basis. Different letters in a column indicate significant differences, as determined by Student–Newman–Keul test (*p* < 0.05). CFA—16% soybean meal + 22% a mixture of fine wheat and wheat flour + 15% broken rice+ 25% maize + 5% bergafats + 1% lysine amino acid +1% DCP + 1% methionine + 1% vitamin-mineral premix + 1% lime + 15% fish meal; CFB—98% cassava leaves + 1% sugar + 1% baking powder; CFC—20% silver leaf desmodium + 20% wheat bran + 40% cowpea bran + 20% maize bran; CFD—30% tropical African morning glory, 30% maize bran, 20% navy bean bran, 10% maize germ, 10% rice bran; CFE—20% tropical African morning glory, CFF—30% tropical African morning glory + 40% cassava leaves powder + 20% cowpea bran + 10% of navy bean bran; CFG—20% tropical African morning glory, 20% gallant soldier, 15% cassava leaves, 20% cowpea bran, 10% navy bean bran, 10% maize bran, 5% wheat bran; CFH—30% cassava leaves + gallant soldier + 20% cowpea bran + 10% tropical African morning glory + 10% taro leaves; CFI—99% wheat bran and 1% baking powder; CFJ—33% maize bran, 33% cassava tuber bran, 33% wheat bran, 1% baking powder; DCP is dicalcium phosphate.

### 3.5. Proximates Values of Gryllus madagascarensis Reared on Different Formulated Feeds and Reference Feed

Proximates of *G. madagascarensis* reared on the different formulated feeds and chicken feed are shown in Table 5. Feeds had a significant effect on the proximate content of *G. madagascarensis*. Moisture contents of *G. madagascarensis* adults ranged from 4.26–8.19% and varied significantly between feeds (Table 5). Crickets raised on CFC, CFD, and CFG had the least moisture content, similar to that of the control, compared with crickets fed on other feeds. Low moisture content, such as in crickets fed feeds CFC, CFD, CFG, and the reference (CFA), increases the shelf life of crickets prepared for consumption as compared with crickets with higher moisture content, such as those fed CFE. The protein content of *G. madagascarensis* ranged between 50.2 and 66.8% (Table 5). It differed across the feeds, with *G. madagascarensis* reared on feed CFF having the highest protein content, followed by crickets fed CFG, with crickets fed CFJ containing the least protein. The crude fat contents were 6.23–19.43% and varied among feeds (Table 5). Crickets reared on CFC had the highest quantity of crude fats and energy compared with crickets fed on other feeds. Crickets reared on CFF contained the least crude fat. Similarly, the ash contents of adult *G. madagascarensis* differed significantly among crickets fed on different feeds and were between 4.3 and 6.0% (Table 5). *Gryllus madagascarensis* fed on CFJ had the highest ash content compared with crickets with other feeds. Fibre contents of crickets raised on different feeds ranged between 8.8 and 13.5% (Table 5). *Gryllus madagascarensis* reared on CFG contained the highest fiber content. *Gryllus madagascarensis* grown on feed CFI were the highest in carbohydrates, while those reared on CFE were the lowest in carbohydrates. Crickets fed CFC had the highest energy compared with those fed on other feeds.

### 3.6. Mineral Elements in Gryllus madagascarensis Cricket Fed on Different Feeds

The mineral contents of *G. madagascarensis* fed different formulated feeds and chicken feed per 100 g dry weight are shown in Table 6. Diets had a significant influence on the mineral elements detected in *G. madagascarensis*. All crickets fed on different diets contained predominantly potassium (accounting for 48.1–53.9% of all minerals), followed by phosphorus (28.7–31.2%), sodium (9.8–15.2%), calcium (3.4–6.5%), magnesium (3.9–4.4%), zinc (0.6–0.8%), iron (0.04–0.8%), manganese (0.1–0.2%), and copper (0.06–0.1%). Crickets fed CFG exhibited the highest magnesium, calcium, iron, phosphorus, zinc, and potassium content. Crickets reared on all feeds exhibited higher calcium than magnesium content. Crickets fed CFD and CFG exhibited the highest manganese compared with crickets reared on feeds. Sodium content was also distributed differently among crickets reared on different feeds, with the highest amounts being detected in crickets fed on feed CFF (up to 1.5-fold higher than the others).

### 3.7. Fatty Acid Content in Gryllus madagascarensis Cricket Fed on Different Feeds

The fatty acid contents of *G. madagascarensis* fed different formulated feeds and reference feed per 100 g dry weight are shown in Table 7. Feeds had a significant impact on the lipid content of *G. madagascarensis*. *Gryllus madagascarensis* fed on various feeds contained predominantly linoleic acid (ranging from 32.2 to 48.2% of the total fats), followed by oleic acid (18.4–39.0%), palmitic acid (7.5–19.7%), stearic acid (7.3–16.3%), behenic acid (0.3–7.9%), lignoceric acid (0.3–3.4%), arachidic acid (0.8–2.9%), myristic acid (0.3–1.4%), margaric acid (0.3–1.4%), alpha-linolenic acid (0.7–1.0%), and eicosapentaenoic acid (0.2–0.3%). Crickets fed CFC had the highest linoleic acid, stearic acid, and margaric acid contents compared with other formulated feeds and the reference feed. Crickets fed CFG exhibited the highest alpha-linolenic acid, myristic acid, and pentadecanoic acid levels compared with the rest of the feeds. The highest palmitic acid content was observed in crickets fed on CFF, while the highest lignoceric acid content was detected in crickets reared on CFI. The highest quantity of arachidic acid was recorded on crickets reared on CFJ. Eicosapentaenoic acid was only detected on crickets reared on CFF and CFG. Crickets reared on CFJ had the lowest ratio of polyunsaturated fatty acid to saturated fatty acids, followed by those reared on CFG, and the highest ratio was noted in crickets fed CFE.

### 3.8. Cost of Feeds Used

All formulated feeds tested were significantly less expensive than the chicken feed. CFH feed was the least costly (USD 0.141/kg), followed by CFG and CFF (USD 0.165/kg, respectively), then CFC (USD 0.188/kg), CFD (USD 0.212/kg), CFE (USD 0.235/kg), CFB (USD 0.251/kg), and CFI and CFJ (USD 0.253/kg, respectively), while the control (chicken feed) (CFA) was the most expensive (USD 0.893/kg) (Table 8).

## 4. Discussion

The increasing cost of the chicken feed used to feed crickets has become a major obstacle to farming crickets for many households. Researchers worldwide are urged to develop alternatives to chicken feed for cricket culture. One of the available options is to use organic side streams as feed for crickets. Feeds formulated from weeds and agro-byproducts are being investigated as feed for crickets for human and animal consumption [37]. The present research revealed that feeds formulated from weed and agro-byproducts are promising alternatives to replace chicken feed for farming *G. madagascarensis* in small-scale and large-scale cricket farm conditions. Feed composition has been reported to influence cricket feed consumption, phenotype, fecundity, and nutrition content [38]. Our study findings showed variations in development growth, survival, reproductive performance, feed efficiency, the nutrition content of *G. madagascarensis,* and cost of production when the insects are reared on formulated feeds from single-plant product powders formulated from weeds and agro-byproducts.

The current study has demonstrated that development time was impacted by the type of feed used to rear *G. madagascarensis*. Our study revealed that *G. madagascarensis* grown on the reference feed took 29 days (4 weeks) to complete the nymphal cycle through the eclosion period to adult crickets. The short development time recorded differed from the seven weeks reported by [17], who grew *G. madagascarensis* collected from the wild on a reference feed of 20% protein content. The variation in development could be due to the differences in the nutrition content of the feeds and rearing room conditions such as humidity, temperature, and light period. On the other hand, our study has shown that *G. madagascarensis* nymphs reared on nine different formulated feeds took ~28 to 41 days (4 to 6 weeks) to reach the adult stage. The difference recorded was due to differences in the nutrient content of the formulated feeds. For instance, the percentage of protein in cricket feeds has been reported to influence their development [39,40,41]. Development time decreases when crickets are reared on high-protein feeds [3,19]. The shortest development time was associated with CFC, which has a protein content of 24.5%, and CFE, consisting of 23.5% protein. The longest development time was recorded in CFH, which has a protein level of 13.5%. In the current study, higher protein levels did not drastically shorten the development time, as seen with *G. madagascarensis* fed CFB (protein content 25.0%) and the reference feed (protein content 28.0%). Previous studies also reported that speeding up development in crickets requires a diet that has more carbohydrates and less fat [41]. In the present study, CFC, with 27.8% carbohydrates and 3.8% fat, and CFE, with 42.2% carbohydrate and 2.1% fat, led to the shortest development time relative to the reference feed, with a carbohydrate content of 43.7% and 3.4% fat. By contrast, CFJ, linked to the longest development time, had the highest levels of carbohydrates (56.6%) and the lowest level of crude fat (1.3%). This indicates that to achieve faster development, these crickets require a feed with an optimum ratio of protein to carbohydrate to fat. Proteins in a feed are important since they contain the amino acids required for the synthesis of cricket proteins [41]. On the other hand, carbohydrates and fats provide energy, which is required for molting, searching for food and water, mating, and other metabolic activities. *Gryllus madagascarensis* nymph development time to the adult stage in the current study was shorter (28–41 days) than the development time of nymphs of *Gryllus bimaculatus* (36–48 days), *Gryllodes sigillatus* (34–60 days), *Acheta domesticus* (42–49 days), *Gryllus assimilis* (44–64 days), and *Scapsipedus icipe* (65–107 days) [2,19,39,42,43,44].

Feed is reported to impact the survival rate of crickets [3,45]. In the current study, *G. madagascarensis* reared on the formulated and reference feeds showed a survival rate ranging between 66 and 98%. However, the highest survival rate (98%) occurred in *G. madagascarensis* reared on CFG, CFE, and CFC feeds, as well as the reference feed (CFA). The lowest survival rate was reported in crickets raised on CFD (66%). The differences in the survival rate of *G. madagascarensis* observed in the current study could be linked to the differences recorded on the formulated and the reference feed. Previous studies reported that crickets reared on formulated diets, which were well balanced in nutrients, experienced reduced mortality [27,45,46]. Moreover, similar reports indicated that when crickets were grown on low-quality formulated feeds, nymph mortality increased because their nutritional needs were not met [45,47]. Therefore, the higher survivorship observed with feeds CFG, CFE, and CFC, having a protein content ranging between 21.5 and 24.4%, indicated that these feeds possessed optimum percentages of nutrients required by the *G. madagascarensis* cricket compared with other feeds. The survival rate on CFG, CFE, and CFC was similar to that of the reference diet. Our findings are consistent with previous research, which indicated that crickets such as *A. domesticus* reared on feeds with over 20% protein tended to have high survival rates of up to 96% due to decreased damage during development [20,41,46]. Apart from protein, crickets also require carbohydrates, fiber, fat, and mineral elements for survival. The current studies indicated that *G. madagascarensis* reared on feeds with carbohydrates (27.8–42.2%), fat (2.1–6.3%), and fiber (10.6–22.7%) led to the highest cricket survival rates. Our results are consistent with previous studies that indicated that crickets required carbohydrates ranging from 32 to 47% and fat (3.2–5.2%) to have a high survival rate. Additionally, crickets need optimal mineral elements to survive. From the present study, the highest survivorship was recorded in *G. madagascarensis* reared on formulated feed CFG, CFE, and CFC, with levels of iron ranging from 171.1 to 881.4 mg/kgDM, copper (6.8–9.7 mg/kgDM), zinc (37.1–64.2 mg/kgDM), manganese (35.9–79.3 mg/kgDM), sodium (1939.3–2399.9 mg/kgDM), magnesium (0.2%), calcium (0.9–1.1%), phosphorus (0.2–0.3%), and potassium (1.2–1.6%) compared with other formulated feeds and the reference feed. Minerals such as calcium and magnesium play a vital role in insect physiology. To survive, insects require optimum amounts of sodium and iron, whereas excess or inadequate amounts of these two minerals lead to mortality [48,49]. On the other hand, when insects like ants are fed on feeds with higher protein content, there is a decreased survival rate due to high mortality [50]. Therefore, while preparing feeds for crickets, the ingredients should be mixed in a manner that results in optimal nutrient levels that will enhance their survival. The weeds and agro-byproducts used to formulate CFG, CFE, and CFC (heterogenous feeds) show their potential as substitutions for chicken feed in cricket production.

Feeds are reported to significantly affect cricket body mass and length [40,51]. Determination of the feed with optimum nutrients to promote large, heavy bodies in *G. madagascarensis* is a paramount factor for boosting production, profits, and sustainable farming of this cricket. The results of the present study demonstrate that *G. madagascarensis* body length and wet body mass were greater in crickets fed on feed with moderately high quantities of protein than those fed on feeds where the protein level was too high or too low. For instance, the result of the current study demonstrates that the heaviest body masses and largest body sizes were attained by crickets fed CFG, which has a protein content of 21.5%—which is intermediate compared with other formulated feeds and the reference feed. The body mass and length of the crickets decreased when the insects were fed a diet either too high in protein, as with CFA (reference feed), which has a protein content of 28.0%, or too low in protein, such as CFJ (13.5% protein). Crickets fed on feeds with optimum nutrients result in crickets whose body length doubles during the juvenile molting, resulting in an exponential expansion of length from one juvenile stage to the other, until the last juvenile stage, where the body content accumulates [19]. From our findings, CFG feed seems to contain the optimum combination of nutrients required to grow the largest and heaviest *G. madagascarensis*, the ultimate goal of cricket farmers. The results of the current study corroborate early reports that suggest that feed with optimum nutrients leads to an increase in feed consumption in crickets, which in turn leads to an increased metabolic rate and body content accumulation [38].

The feed conversion ratio (FCR) is a good indicator of the feed quality and efficiency of farmed crickets to make use of it. Previous studies have reported crickets to be high-feed converters [14]. Comparing the impact of different formulated feeds on the FCR of *G. madagascarensis*, our findings revealed that when the protein and mineral content of the feed is increased to the optimum level, the FCR decreases. *Gryllus madagascarensis* fed on feeds CFG, CFE, and CFC had the lowest FCR compared with other formulated feeds and the reference feed. The small FCR showed by crickets fed on CFG, CFE, and CFC demonstrated that they converted the feed offered to them efficiently into biomass. The small FCR also shows that CFG, CFE, and CFC had high nutrition quality compared with other formulated and reference feeds. Crickets fed on CFJ, with its too-low protein content, and CFA, with its too-high protein content, exhibited high FCR, indicating that these groups were less efficient at converting feed to body mass. The FCR reported in this study is within the range of the FCR reported previously [14,44].

The type of feed influences the cost of feeding crickets [14]. Many cricket farmers desire to use cheaper feeds to obtain higher returns on their investment. One way to achieve this is by determining the cost of producing one kilogram of live crickets. In the present study, the cost of feeding per kg live mass gain in *G. madagascarensis* reared on feed CFH and CFG was significantly (7-fold) lower than feed CFJ and the reference feed. However, crickets fed CFH recorded a lower mean body weight and consumed more feed than crickets fed CFG. This has huge implications for income, while feeding CFG with intermediately high crude protein content may make it somewhat expensive to produce a kilogram of crickets, even though less feed is required than if using CFH. Also, feeding crickets on CFG will lead to a higher yield than other feeds. Therefore, feeding farmed crickets with feed CFG may be cheaper in the long run than using a feed too low or too high in crude protein.

The nutritional content of feed offered to the crickets has been reported to influence their reproductive yield. For instance, crickets fed on feed rich in protein will lay more eggs, have a short preoviposition period, their eggs will take less time to hatch, and egg hatch percentage will be high. In the present study, preoviposition duration, fecundity, egg incubation period, and egg hatchability in *G. madagascarensis* reared on different formulated diets varied across the feeds. In this case, the shortest preoviposition, highest fecundity, shortest egg incubation period, and highest hatchability were recorded with *G. madagascarensis* females fed on CFG. This was likely caused by the higher protein content, moderately high carbohydrate content, high ash content, high fiber content, high iron, high sodium, high calcium, and high phosphorous of CFG. This combination of nutrients has been reported to lower preoviposition time and egg incubation period and increase egg laying and egg hatchability in other cricket species [19,41]. On the other hand, the lowest preoviposition and fecundity, longest egg incubation period, and lowest egg hatchability were recorded in females fed CFJ. This feed contained the lowest quantity of protein, was high in carbohydrates, and was low in ash, iron, phosphorus, manganese, sodium, and calcium content, which corroborates previous reports [52]. Further, studies on *G. assimilis* and *G. sigillatus* have shown that crickets fed on diets rich in high-quality proteins, carbohydrates, iron, and phosphorus will have larger, quicker-maturing ovaries able to accommodate more eggs than crickets fed low-quality diets [41]. It is important to note that *G. madagascarensis*, which produces 1130.6–2554.10 eggs, is comparable in fecundity to *G. bimaculatus*, *S. icipe*, *G. assimilis,* and *A. domesticus* reared on weeds and agro-byproducts [53].

Different studies have reported feeds to have varying influences on the nutrition content of crickets [13,16]. Crickets fed on highly nutritious feeds have been reported to be a good source of protein, fat, and fiber (chitin). Our results show that *G. madagascrensis* reared on feeds with varying nutrition content led to variation in the nutrition content of the cricket across the formulated feeds and the reference diet. Additionally, the nutritional analysis of *G. madagascarensis* fed on formulated feeds revealed that protein was the major component, averaging 66.78%, followed by fats, fiber, moisture, ash, and carbohydrates. The nutrient content obtained from the crickets fed various feeds in our study is in tandem with previous studies, where reported protein varied from 59.70 to 75.00%, carbohydrate from 1.13 to 11.90%, fiber from 4.60 to 8.68%, fat from 8.00 to 23.80%, and ash from 3.79 to 5.40% [54]. Crickets fed CFF (22.5% protein) had the highest protein content, followed by crickets fed CFG (21.5% protein), while crickets fed CFJ (13.5% protein) had the least nutrient content. Crickets reared on CFC had the highest fat content compared with the other feeds. Fat is a source of metabolic energy for human beings. Fat from crickets can be used as a raw material for industries manufacturing cooking oils and cosmetics. Therefore, farmers who want to produce crickets for fat extraction should feed CFC to their crickets. The findings demonstrate that crickets reared on CFG resulted in crickets with the highest fiber level, lacking in animal protein sources. Cricket fiber comes in the form of chitin, which has been reported to have human health benefits. For instance, chitin in crickets has been reported to serve as a prebiotic component enhancing the growth of *Bifidobacterium animalis* in the digestive system of human beings, required for digestion and assimilation of food [55,56]. Moreover, chitin in edible crickets has been reported to reduce swelling in the gastrointestinal lining and improve the function of the heart [56]. Additionally, chitin is reported to be an immune booster of eosinophils, macrophages, and T helper cells found in the lungs and gut [57].

Feed is reported to influence the level of mineral elements in crickets [6]. Minerals are important for the normal functioning of the cricket body [58]. *Gryllus madagascarensis* reared on the nine formulated feeds and reference feeds varied in their content of mineral elements. This was due to differences in the nutrition content of various weeds and agro-byproducts used in formulating the feeds. *Gryllus madagascarensis* reared on CFG with seven mixed ingredients was richest in potassium, calcium, magnesium, phosphorus, iron, zinc, and manganese compared with crickets reared on other formulated feeds. Crickets reared on CFI exhibited the lowest mineral element content. This could be because CFI is nearly homogeneous since it consists of one agro-byproduct and baking powder. Previous studies have demonstrated that when crickets are fed on a single-plant product, they always suffer deficiencies in some mineral elements [19]. This implies that no single-plant product can supply sufficient mineral elements to meet the requirements of crickets. To overcome the deficiency, several plant products should be blended to attain a feed with diversified mineral elements, as found in CFG. The current study further demonstrated that potassium was the major mineral element, followed by phosphorus and sodium in *G. madagascarensis*. This finding is similar to a previous study conducted on *A. domesticus* [14].

The current study revealed that when *G. madagascarensis* was fed different feed formulations, the resulting adult crickets varied in their fatty acid content. *Gryllus madagascarensis* grown on CFG and CFC were the richest in fatty acids. Linoleic acid formed the major fatty acid component, followed by oleic and palmitic acid, in crickets fed the formulated and the reference feeds. This result differs from the findings in [58], which reported oleic acid as the major fatty acid, followed by linoleic and palmitic acid in *A. domesticus*. Even when fed on different diets, *G. madagascarensis* was shown to be a good supplier of alpha-linolenic acid (omega-3 fatty acids) and linoleic acid (omega-6 fatty acids), both essential fatty acids. The level of essential fatty acids (EFAs) obtained in this study was comparable to the results reported by [8] for *A. domesticus.* On the other hand, the reported fatty acids in this study were higher than those found in *G. bimaculatus* (4.25–27.92%) and *G. assimilis* (26.81%) [59]. Essential fatty acids in edible crickets are important as they reflect the quality of insect oils [60]. The ratio of polyunsaturated fatty acids (PUFA) to saturated fatty acids (SFA) varied according to the feed consumed by *G. madagascarensis*. In our study, the ratio of PUFA to SFA was low and within the range of the 5:1 ratio recommended for health [61]. These low PUFA-to-SFA ratios help prevent obesity, asthma, high blood pressure, *Diabetes mellitus*, and some cancers [61].

Regarding the cost of formulating one kilogram of the feeds, the current study demonstrated that the formulated feeds made from single-plant products from weeds and agro-byproducts incurred different costs. Formulated feeds were four to six times cheaper than the reference diet. CFG and CFF feeds were the most affordable to make compared with other feeds. This could be because using several single-plant products in feed formulation requires less of a particular agro-byproduct, especially those that might be expensive, reducing the final cost of the feed. The reference feed was the most expensive, making it the most inaccessible, unaffordable, and unsustainable feed choice for resource-poor farmers. By contrast, feeds formulated from weeds and agro-byproducts provide a cost-effective benefit but are still able to satisfy the dietary demands of *G. madagascarensis*. Despite formulated feeds varying in their nutritional components, they generally provide enough nutrients for *G. madagascarensis*. The global search for cheap feed suitable for farming edible crickets has been triggered by the extremely high cost of chicken feed, which has discouraged many people from farming crickets [62]. We recommend that cricket farmers in Madagascar, a country rich in a variety of weeds and agro-byproducts, make use of these locally available and less expensive ingredients to develop feeds for their crickets while reducing the cost of cricket farming. Since supplies of weeds and agro-byproducts have seasonal limitations, cricket farmers are advised to harvest them in season, rinse them with clean water, sun dry them, then mill them into powders and pack them in hermetically sealed bags before storing them for future use. Further, weeds and agro-byproducts are bulky, requiring a lot of space for drying and storage in the case of large-scale cricket farming. To overcome such limitations, large-scale farmers can construct stacked wooden driers to make use of the sun, especially in areas where space for drying harvested weeds is limited. Moreover, large-scale farmers can construct simple barns on the farm to store the milled weeds and agro-byproducts. Wide adoption of this method can be encouraged with awareness campaigns among cricket farmers.

## 5. Conclusions

This study is the first of its kind to report on the use of inexpensive, locally available, and sustainable weeds and agro-byproducts to formulate an optimal and affordable feed for farming *G. madagascarensis*. The current study has identified feed CFG as the most optimal for farming *G. madagascarensis*, followed by CFE and CFC. All three of these feeds support rapid development, very high survival rates, body masses and sizes, fecundity, and egg hatchability in *G. madagascarensis*. Further, crickets fed on these feeds produced insects rich in protein, lipids, fiber, mineral salts, and fatty acids, which can help mitigate the malnutrition rampant in Madagascar. When the formulated feeds and reference feed were ranked in terms of cricket performance, CFG, CFE, and CFC emerged as the three top performers, in that order. CFG, in particular, resulted in a high survival rate, large and heavy crickets, low feed conversion ratio, low cost of producing one kilogram of crickets, low cost of making one kilogram of the feed, short preoviposition period, and high fecundity of female crickets. Moreover, CFG produced crickets with high levels of minerals such as iron and zinc, which are often deficient in children and pregnant women. Moreover, crickets fed CFG produced essential fatty acids, which are required for brain development in school-going children. The efficiency of CFG was attributed to its high nutrient content, which is related to improved growth and reproductive performance in insects. The cost–benefit ratio and return on investment in the feed make it a suitable and promising alternative that would be affordable to everyone, including the resource-poor and vulnerable portions of the communities where *G. madagascarensis* is widely consumed. Equally important, the weeds and agro-byproducts from which CFG is developed are found across Madagascar, making this formulated feed a practical substitute for chicken feed. As this is the first study of its kind on *G. madagascarensis*, these findings offer groundbreaking information that may be useful when designing feed for farming *G. madagascarensis* in Madagascar and other parts of the world. These findings also contribute to the body of knowledge needed to farm crickets at a large scale to mitigate food insecurity and malnutrition around the world. Our findings further reveal that *G. madagascarensis* is tolerant to feeds with different nutrient content blended from a variety of weeds and agro-byproducts. Accompanying the idea that the human population is to reach about 10 billion people by the year 2050, *G. madagascarensis* fed on feeds formulated from weeds and agro-byproducts forms a promising candidate for protein production. Further optimization of this feed would enable the production of *G. madagascarensis* year round. This will help overcome challenges and bridge the gap of unsustainable dependence on expensive and unsustainable chicken feed to rear crickets. Further studies are recommended to determine the effect of CFG on the amino acid quality and sensory properties of *G. madagascarensis* to improve the nutrition and health of consumers. Further studies also need to be conducted to explore the use of other weeds and agro-byproducts in developing different cricket feeds that will facilitate maximum development, survival, growth, reproduction, and nutrition of *G. madagascarensis* to replace chicken feed to farm crickets across Africa. Lastly, the use of weeds and agro-byproducts in developing cricket feeds could improve the environmental sustainability of cricket feeds and cricket farming and promote a circular economy.

## Figures and Tables

**Table 1:** Formulated feeds and control used for rearing *Gryllus madagascarensis*.

Feed	Ingredients
CFA (chicken feed) (control)	16% soybean meal, 22% a mixture of fine wheat and wheat flour, 15% broken rice, 25% maize, 5% bergafats, 1% lysine amino acid, 1% DCP, 1% methionine, 1% vitamin-mineral premix, 1% lime, 15% fish meal
CFB	98% cassava leaves, 1% sugar and 1% baking powder
CFC	20% silver leaf *Desmodium*, 20% wheat bran, 20% maize bran and 40% cowpea bran
CFD	30% tropical African morning glory, 30% maize bran, 20% navy bean bran, 10% maize germ, 10% rice bran
CFE	20% tropical African morning glory, 20% cassava leaves, 20% maize bran, 10% rice bran, 10% cowpea bran, 20% navy bean bran
CFF	30% tropical African morning glory, 40% cassava leaves powder, 20% cowpeas bran and 10% of navy bean bran
CFG	20% tropical African morning glory, 20% gallant soldier, 15% cassava leaves, 20% cowpea bran, 10% navy bean bran, 10% maize bran, 5% wheat bran
CFH	30% cassava leaves, 30% gallant soldier, 20% cowpea bran, 10% tropical African morning glory, 10% taro leaves
CFI	99% wheat bran and 1% baking powder
CFJ	33% maize bran, 33% cassava tuber bran, 33% wheat bran, 1% baking powder

Note: CFA (Chicken feed), CFB (Combined feed B), CFC (Combined feed C), CFD (Combined feed D), CFE (Combined feed E), CFF (Combined feed F), CFG (Combined feed G), CFH (Combined feed H), CFI (Combined feed I), CFJ (Combined feed J), and DCP means dicalcium phosphate.

**Table 5:** Proximate content in *Gryllus madagascarensis* cricket fed on different feeds (% dry matter).

*Gryllus madagascarensis* Fed	Moisture	Crude Protein	Crude Fat	Ash	Crude Fiber	Carbohydrate	Energy (kcal/kg)
CFA	4.74 ± 0.25 b	59.68 ± 0.29 d	18.72 ± 0.40 ab	4.26 ± 0.09 a	12.52 ± 0.58 b	0.08 ± 0.01 c	4073.88 ± 43.94 b
CFB	7.56 ± 0.35 d	54.41 ± 1.01 f	17.24 ± 0.80 c	4.43 ± 0.15 bc	10.14 ± 0.12 d	6.22 ± 0.03 b	3950.28 ± 90.30 bcd
CFC	4.26 ± 0.05 a	57.35 ± 1.46 e	19.43 ± 1.23 a	4.46 ± 0.03 bc	8.80 ± 0.59 e	5.70 ± 1.27 b	4280 ± 98.40 a
CFD	4.87 ± 0.31 b	62.60 ± 0.38 c	16.19 ± 0.51 c	4.58 ± 0.20 b	10.05 ± 0.12 d	1.71 ± 0.23 c	4029.37 ± 51.30 bc
CFE	8.19 ± 0.23 e	60.70 ± 1.02 d	13.46 ± 0.63 d	4.96 ± 0.03 d	11.54 ± 0.34 c	1.15 ± 0.38 c	3720.17 ± 42.24 e
CFF	6.79 ± 0.39 c	66.76 ± 0.04 a	6.23 ± 0.44 f	5.34 ± 0.17 e	12.79 ± 0.10 ab	2.09 ± 0.72 c	3314.70 ± 10.91 g
CFG	5.05 ± 0.25 b	64.74 ± 0.12 b	9.89 ± 0.37 e	4.84 ± 0.14 cd	13.51 ± 1.10 a	1.97 ± 0.95 c	3558.20 ± 75.16 f
CFH	7.49 ± 0.16 d	57.03 ± 1.15 e	17.64 ± 0.38 bc	5.18 ± 0.04 ef	9.94 ± 0.05 d	2.72 ± 1.60 c	3977.43 ± 14.01 bcd
CFI	7.52 ± 0.12 d	51.08 ± 0.69 g	16.08 ± 0.86 c	5.14 ± 0.12 ef	10.55 ± 0.19 d	9.63 ± 1.12 a	3874.80 ± 49.57 d
CFJ	7.44 ± 0.16 d	50.19 ± 0.54 g	17.13 ± 0.75 b	6.02 ± 0.10 f	9.88 ± 0.34 d	9.34 ± 1.38 a	3922.73 ± 36.85 cd
F_9,20_	106.60	140.70	111.00	46.14	32.35	27.81	67.14
*p*	<0.001	<0.001	<0.001	<0.001	<0.001	<0.001	<0.001

The results are expressed as mean values ± standard deviation of three determinations on a dry matter (DM) basis. Different letters in a column indicate significant differences, as determined by Student–Newman–Keul test (*p* < 0.05). CFA—16% soybean meal + 22% a mixture of fine wheat and wheat flour + 15% broken rice+ 25% maize + 5% bergafats + 1% lysine amino acid +1% DCP + 1% methionine + 1% vitamin-mineral premix + 1% lime + 15% fish meal; CFB—98% cassava leaves + 1% sugar + 1% baking powder; CFC—20% silver leaf desmodium + 20% wheat bran + 40% cowpea bran + 20% maize bran; CFD—30% tropical African morning glory, 30% maize bran, 20% navy bean bran, 10% maize germ, 10% rice bran; CFE—20% tropical African morning glory, CFF—30% tropical African morning glory + 40% cassava leaves powder + 20% cowpea bran + 10% of navy bean bran; CFG—20% tropical African morning glory, 20% gallant soldier, 15% cassava leaves, 20% cowpea bran, 10% navy bean bran, 10% maize bran, 5% wheat bran; CFH—30% cassava leaves + gallant soldier + 20% cowpea bran + 10% tropical African morning glory + 10% taro leaves; CFI—99% wheat bran and 1% baking powder; CFJ—33% maize bran, 33% cassava tuber bran, 33% wheat bran, 1% baking powder; DCP is dicalcium phosphate.

**Table 6:** Mineral elements in *Gryllus madagascarensis* cricket fed on different feeds compared with the required daily intake.

*Gryllus madagascarensis* Fed	Mineral Elements (mg/100 g)								
	Magnesium (Mg)	Iron (Fe)	Calcium (Ca)	Copper (Cu)	Phosphorus (P)	Zinc (Zn)	Potassium (K)	Manganese (Mn)	Sodium (Na)
CFA	80.60 ± 8.92 c	0.82 ± 0.08 f	90.89 ± 7.23 e	2.67 ± 0.10 a	626.58 ± 0.01 d	14.31 ± 1.20 d	1053.56 ± 65.40 cd	3.16 ± 0.20 d	327.73 ± 24.62 c
CFB	76.56 ± 2.67 c	7.57 ± 0.56 cd	93.78 ± 5.10 e	1.83 ± 0.03 e	566.59 ± 2.12 f	10.75 ± 0.25 f	990.65 ± 3.41 d	2.37 ± 0.03 e	264.70 ± 5.77 d
CFC	102.66 ± 4.63 ab	8.13 ± 0.75 c	138.03 ± 5.73 c	2.36 ± 0.23 b	765.76 ± 0.01 b	16.89 ± 0.14 c	1102.09 ± 35.20 bc	2.39 ± 0.01 e	331.26 ± 11.56 c
CFD	93.51 ± 7.96 b	6.01 ± 0.75 d	112.89 ± 8.61 d	2.20 ± 0.14 bc	727.08 ± 0.01 e	13.56 ± 0.47 d	1132.72 ± 25.84 bc	4.08 ± 0.25 a	323.55 ± 13.95 c
CFE	102.66 ± 4.63 ab	11.17 ± 0.89 b	151.18 ± 0.66 b	1.89 ± 0.16 de	730.30 ± 0.01 c	17.03 ± 0.99 c	1073.24 ± 73.51 c	3.31 ± 0.22 cd	352.46 ± 8.88 b
CFF	96.32 ± 1.07 b	6.64 ± 0.38 cd	160.08 ± 4.20 b	2.40 ± 0.09 b	731.85 ± 0.02 c	18.98 ± 0.37 b	1166.44 ± 14.43 ab	3.67 ± 0.01 b	392.74 ± 12.14 a
CFG	108.14 ± 7.06 a	22.47 ± 2.07 a	172.40 ± 8.20 a	2.07 ± 0.01 cd	781.95 ± 1.00 a	21.46 ± 0.72 a	1232.04 ± 3.54 a	3.93 ± 0.01 a	358.12 ± 0.19 b
CFH	79.21 ± 0.57 c	8.14 ± 0.16 c	101.12 ± 1.81 e	1.35 ± 0.01 f	577.21 ± 0.01 e	11.85 ± 0.25 e	1214 ± 7.01 a	3.48 ± 0.01 bc	275.32 ± 12.96 d
CFI	73.54 ± 3.19 c	4.78 ± 0.20 e	63.88 ± 2.49 f	1.75 ± 0.04 e	552.68 ± 8.72 g	10.42 ± 0.31 f	825.56 ± 26.14 e	1.81 ± 0.05 f	182.93 ± 4.16 e
CFJ	71.48 ± 3.83 c	4.63 ± 0.18 e	61.91 ± 2.51 f	1.70 ± 0.05 e	525.68 ± 4.13 h	10.01 ± 0.63 f	975.76 ± 51.31 e	1.78 ± 0.01 f	179.13 ± 1.28 e
F_9,20_	22.58	147.2	160.8	32.73	2940	118.4	44.27	168.8	121.3
*p*	<0.001	<0.001	<0.001	<0.001	<0.001	<0.001	<0.001	<0.001	<0.001

The results are expressed as mean values ± standard deviation of three determinations on a dry matter (DM) basis. Different letters in a column indicate significant differences, as determined by Student–Newman–Keul test (*p* < 0.05). CFA—16% soybean meal + 22% a mixture of fine wheat and wheat flour + 15% broken rice+ 25% maize + 5% bergafats + 1% lysine amino acid +1% DCP + 1% methionine + 1% vitamin-mineral premix + 1% lime + 15% fish meal; CFB—98% cassava leaves + 1% sugar + 1% baking powder; CFC—20% silver leaf desmodium + 20% wheat bran + 40% cowpea bran + 20% maize bran; CFD—30% tropical African morning glory, 30% maize bran, 20% navy bean bran, 10% maize germ, 10% rice bran; CFE—20% tropical African morning glory, CFF—30% tropical African morning glory + 40% cassava leaves powder + 20% cowpea bran + 10% of navy bean bran; CFG—20% tropical African morning glory, 20% gallant soldier, 15% cassava leaves, 20% cowpea bran, 10% navy bean bran, 10% maize bran, 5% wheat bran; CFH—30% cassava leaves + gallant soldier + 20% cowpea bran + 10% tropical African morning glory + 10% taro leaves; CFI—99% wheat bran and 1% baking powder; CFJ—33% maize bran, 33% cassava tuber bran, 33% wheat bran, 1% baking powder; DCP is dicalcium phosphate.

**Table 7:** Fatty acids content (%) of oils extracted from *Gryllus madagascarensis* crickets fed different feeds.

FAMEs	Corresponding Fatty Acid	ω-n (Δn)	CFA	CFB	CFC	CFD	CFE	CFF	CFG	CFH	CFI	CFJ	F_9,20_	*p*-Value
Methyl dodecanoate	Lauric acid	C12:0	0.36 ± 0.02 a	0.05 ± 0.00 e	0.09 ± 0.01 d	0.34 ± 0.04 a	ND	ND	0.14 ± 0.01 bc	0.16 ± 0.02 b	0.11 ± 0.01 cd	ND	93.64	0.001
Methyl tetradecanoate	Myristic acid	C14:0	0.66 ± 0.02 c	0.29 ± 0.02 b	1.32 ± 0.05 a	0.89 ± 0.12 b	1.08 ± 0.06 b	0.58 ± 0.07 cd	1.41 ± 0.14 a	0.32 ± 0.02 e	0.48 ± 0.01 cde	0.40 ± 0.06 de	41.10	0.001
Methyl Pentadecanoate	Pentadecanoic acid	C15:0	0.10 ± 0.01 c	0.26 ± 0.07 b	0.43 ± 0.07 a	0.22 ± 0.03 b	0.14 ± 0.03 c	ND	0.46 ± 0.03 a	0.29 ± 0.01 b	0.07 ± 0.02 c	0.22 ± 0.02 b	38.11	0.001
Methyl hexadecanoate	Palmitic acid	C16:0	10.60 ± 0.55 e	11.59 ± 0.93 de	7.53 ± 0.41 f	15.10 ± 0.67 c	12.88 ± 0.92 d	19.75 ± 0.80 a	17.44 ± 0.85 b	19.66 ± 0.32 a	11.89 ± 0.12 de	19.45 ± 0.54 d	138.40	0.001
Heptadecanoic acid	Margaric acid	C17:0	0.42 ± 0.03 de	1.39 ± 0.35 a	1.40 ± 0.07 a	0.37 ± 0.06 de	0.72 ± 0.08 bc	0.90 ± 0.08 b	1.34 ± 0.06 a	0.47 ± 0.03 cde	0.27 ± 0.04 e	0.65 ± 0.12 bcd	35.15	0.001
Methyl octadecanoate	Stearic acid	C18:0	10.00 ± 0.42 de	10.75 ± 0.33 de	16.31 ± 0.47 a	10.73 ± 0.58 de	9.38 ± 0.75 ef	14.34 ± 0.99 b	10.21 ± 0.98 d	13.27 ± 0.23 c	8.43 ± 0.20 f	10.00 ± 0.42 g	59.17	0.001
Methyl eicosanoate	Arachidic acid	C20:0	1.34 ± 0.01 ef	1.59 ± 0.26 de	2.35 ± 0.15 b	1.96 ± 0.14 c	0.75 ± 0.14 g	2.31 ± 0.17 b	1.85 ± 0.11 cd	0.91 ± 0.10 g	1.20 ± 0.03 f	2.94 ± 0.25 a	60.40	0.001
Methyl docosanoate	Behenic acid	C22:0	0.37 ± 0.05 e	1.08 ± 0.07 c	1.13 ± 0.10 c	0.29 ± 0.03 e	ND	0.75 ± 0.14 d	ND	ND	7.93 ± 0.20 a	4.82 ± 0.32 b	1078.00	0.001
Methyl tetracosanoate	Lignoceric acid	C24:0	1.34 ± 0.02 bc	ND	0.31 ± 0.02 c	1.05 ± 0.13 bc	ND	ND	ND	ND	3.42 ± 0.76 a	2.71 ± 0.25 ab	7.61	0.004
Octadecatrienic acid	Alpha-linolenic acid	C 18:3 (*n*-3)	0.43 ± 0.01 f	0.81 ± 0.01 b	0.72 ± 0.01 c	ND	0.48 ± 0.01 e	0.70 ± 0.01 d	1.01 ± 0.01 a	ND	ND	ND	1578.00	0.001
Eicosapentanoic acid (EPA)	Timnodonic acid	C 20:5 (*n*-3)	ND	ND	ND	ND	ND	0.31 ± 0.01 a	0.20 ± 0.01 b	ND	ND	ND	544.50	0.001
Methyl tetracosanoate	Linoleic acid	C18:2 (*n*-6)	35.69 ± 0.48 f	43.73 ± 0.70 d	48.19 ± 0.21 a	40.21 ± 0.77 e	45.23 ± 0.93 c	40.21 ± 0.77 e	32.22 ± 0.79 g	46.50 ± 0.41 b	46.16 ± 0.24 bc	32.13 ± 0.16 g	338.30	0.001
Methyl (9Z)-Octadecenoate	Oleic acid	C18:1 (*n*-9)	38.95 ± 0.63 a	28.46 ± 0.40 c	20.22 ± 0.37 d	28.84 ± 0.84 c	29.34 ± 0.43 c	20.15 ± 0.76 d	33.72 ± 0.55 b	18.42 ± 0.40 e	20.04 ± 1.84 d	28.65 ± 0.55 c	193.10	0.001
Monounsaturated fatty acids (MUFA)			38.95 ± 0.63 a	28.46 ± 0.40 c	20.22 ± 0.37 d	28.84 ± 0.84 c	29.34 ± 0.43 c	20.15 ± 0.76 d	33.72 ± 0.55 b	18.42 ± 0.40 e	20.04 ± 1.84 d	28.65 ± 0.55 c		
Polyunsaturated fatty acids (PUFA)			36.12 ± 0.51	44.54 ± 0.70	48.91 ± 0.24	40.21 ± 0.77	45.72 ± 0.95	41.22 ± 0.77	33.43 ± 0.85	46.50 ± 0.51	46.16 ± 0.24	32.13 ± 0.16		
Saturated fatty acids (SFA)			24.93 ± 1.18	27.81 ± 1.10	30.87 ± 1.36	30.95 ± 1.77	24.95 ± 1.98	38.63 ± 2.24	32.85 ± 2.18	35.08 ± 0.50	33.82 ± 2.08	39.25 ± 0.71		
PUFA/SFA			1.49	1.57	1.58	1.30	1.83	1.07	1.02	1.33	1.36	0.82		
Essential fatty acids (EFAs)			36.70 ± 0.51	44.52 ± 0.70	48.91 ± 0.24	40.21 ± 0.77	45.72 ± 0.95	40.91 a ± 0.89	33.23 ± 0.82	46.50 ± 0.51	46.16 ± 0.24 b	32.13 ± 0.16		

The results are expressed as mean values ± standard deviation of three determinations on a dry matter (DM) basis. Different letters in a column indicate significant differences, as determined by Student–Newman–Keul test (*p* < 0.05). CFA—16% soybean meal + 22% a mixture of fine wheat and wheat flour + 15% broken rice+ 25% maize + 5% bergafats + 1% lysine amino acid +1% DCP + 1% methionine + 1% vitamin-mineral premix + 1% lime + 15% fish meal; CFB—98% cassava leaves + 1% sugar + 1% baking powder; CFC—20% silver leaf desmodium + 20% wheat bran + 40% cowpea bran + 20% maize bran; CFD—30% tropical African morning glory, 30% maize bran, 20% navy bean bran, 10% maize germ, 10% rice bran; CFE—20% tropical African morning glory, CFF—30% tropical African morning glory + 40% cassava leaves powder + 20% cowpea bran + 10% of navy bean bran; CFG—20% tropical African morning glory, 20% gallant soldier, 15% cassava leaves, 20% cowpea bran, 10% navy bean bran, 10% maize bran, 5% wheat bran; CFH—30% cassava leaves + gallant soldier + 20% cowpea bran + 10% tropical African morning glory + 10% taro leaves; CFI—99% wheat bran and 1% baking powder; CFJ—33% maize bran, 33% cassava tuber bran, 33% wheat bran, 1% baking powder; DCP is dicalcium phosphate and ND means not detected.

**Table 8:** Cost of feeds used for rearing *Gryllus madagascarensis*.

Ingredient Combination	Cost per kg (Malagasy Ariary)	Quantity/kg									
		CFA	CFB	CFC	CFD	CFE	CFF	CFG	CFH	CFI	CFJ
Cowpea bran	1000.00	0.00	0.00	0.40	0.00	0.10	0.20	0.20	0.20	0.00	0.00
Wheat bran	1000.00	0.00	0.00	0.20	0.00	0.20	0.00	0.05	0.00	0.99	0.33
Maize bran	1000.00	0.00	0.00	0.20	0.30	0.20	0.00	0.10	0.00	0.00	0.33
Rice bran	1000.00	0.00	0.00	0.00	0.10	0.10	0.00	0.00	0.00	0.00	0.00
Nelly bean bran	1000.00	0.00	0.00	0.00	0.20	0.20	0.10	0.10	0.00	0.00	0.00
Cassava tuber bran	1000.00	0.00	0.00	0.00	0.00	0.00	0.00	0.00	0.00	0.00	0.33
Cassava leaves powder	1000.00	0.00	0.98	0.00	0.00	0.20	0.40	0.15	0.30	0.00	0.00
Soybean meal	4500.00	0.16	0.00	0.00	0.00	0.00	0.00	0.00	0.00	0.00	0.00
Maize	3000.00	0.25	0.00	0.00	0.10	0.00	0.00	0.00	0.00	0.00	0.00
Wheat pollard	1200.00	0.22	0.00	0.00	0.00	0.00	0.00	0.00	0.00	0.00	0.00
Rice polish	900.00	0.15	0.00	0.00	0.00	0.00	0.00	0.00	0.00	0.00	0.00
Tropical African Morning Glory powder	0.00	0.00	0.00	0.00	0.30	0.20	0.30	0.20	0.10	0.00	0.00
Silver leaf desmodium powder	0.00	0.00	0.00	0.20	0.00	0.00	0.00	0.00	0.00	0.00	0.00
Gallant soldier powder	0.00	0.00	0.00	0.00	0.00	0.00	0.00	0.20	0.30	0.00	0.00
Taro tops powder	1000.00	0.00	0.00	0.00	0.00	0.00	0.00	0.00	0.10	0.00	0.00
Sugar	7500	0.00	0.01	0.00	0.00	0.00	0.00	0.00	0.00	0.00	0.00
Baking powder	8650.50	0.00	0.01	0.00	0.00	0.00	0.00	0.00	0.00	0.01	0.01
Bergafat	12,500.00	0.05	0.00	0.00	0.00	0.00	0.00	0.00	0.00	0.00	0.00
Lysine amino acid	13,500.00	0.01	0.00	0.00	0.00	0.00	0.00	0.00	0.00	0.00	0.00
MDCP	18,000.00	0.01	0.00	0.00	0.00	0.00	0.00	0.00	0.00	0.00	0.00
Methionine	37,500.00	0.01	0.00	0.00	0.00	0.00	0.00	0.00	0.00	0.00	0.00
Vitamin mineral premix	13,500.00	0.01	0.00	0.00	0.00	0.00	0.00	0.00	0.00	0.00	0.00
Lime	4000.00	0.01	0.00	0.00	0.00	0.00	0.00	0.00	0.00	0.00	0.00
Fish meal	4500.00	0.12	0.00	0.00	0.00	0.00	0.00	0.00	0.00	0.00	0.00
Cost per Kg (MGA)		3800.00	1066.50	800.00	900.00	1000	700.00	700.00	600.00	1076.50	1076.50
Cost per Kg (USD)		0.893	0.251	0.188	0.212	0.235	0.165	0.165	0.141	0.253	0.253

The results are expressed on a dry matter (DM) basis. CFA—16% soybean meal + 22% a mixture of fine wheat and wheat flour + 15% broken rice+ 25% maize + 5% bergafats + 1% lysine amino acid +1% DCP + 1% methionine + 1% vitamin-mineral premix + 1% lime + 15% fish meal; CFB—98% cassava leaves + 1% sugar + 1% baking powder; CFC—20% silver leaf desmodium + 20% wheat bran + 40% cowpea bran + 20% maize bran; CFD—30% tropical African morning glory, 30% maize bran, 20% navy bean bran, 10% maize germ, 10% rice bran; CFE—20% tropical African morning glory, CFF—30% tropical African morning glory + 40% cassava leaves powder + 20% cowpea bran + 10% of navy bean bran; CFG—20% tropical African morning glory, 20% gallant soldier, 15% cassava leaves, 20% cowpea bran, 10% navy bean bran, 10% maize bran, 5% wheat bran; CFH—30% cassava leaves + gallant soldier + 20% cowpea bran + 10% tropical African morning glory + 10% taro leaves; CFI—99% wheat bran and 1% baking powder; CFJ—33% maize bran, 33% cassava tuber bran, 33% wheat bran, 1% baking powder; DCP is dicalcium phosphate. Where 0 is indicated in each column, the ingredient was not factored in formulating the feed. Note: 1 USD (US dollar) = 4250 MGA during the study period (2023/2024).

## Data Availability

The original contributions presented in this study are included in the article; further inquiries can be directed to the corresponding author.
